# Usability and Acceptability of a Mobile Comprehensive HIV Prevention App for Men Who Have Sex With Men: A Pilot Study

**DOI:** 10.2196/mhealth.7199

**Published:** 2017-03-09

**Authors:** Patrick S Sullivan, Robert Driggers, Joanne D Stekler, Aaron Siegler, Tamar Goldenberg, Sarah J McDougal, Jason Caucutt, Jeb Jones, Rob Stephenson

**Affiliations:** ^1^ Rollins School of Public Health Department of Epidemiology Emory University Atlanta, GA United States; ^2^ Department of Medicine University of Washington Seattle, WA United States; ^3^ Center for Sexuality and Health Disparities School of Nursing University of Michigan Ann Arbor, MI United States; ^4^ Department of Medicine Department of Medicine University of Washington Seattle, WA United States

**Keywords:** homosexuality, male, mobile applications, pilot projects, sexual minorities, condoms, pre-exposure prophylaxis

## Abstract

**Background:**

Men who have sex with men (MSM) are the group most impacted by the human immunodeficiency virus (HIV) epidemic and the only subgroup in the United States among which new HIV diagnoses are not decreasing. To achieve the US National HIV/AIDS (acquired immunodeficiency syndrome) Strategy goals of reducing new diagnoses by 25%, high (eg, 30-50%) coverage of multiple HIV prevention interventions is needed in both urban and rural areas. Mobile phone “apps” are an important channel through which prevention services could be provided at scale and at low marginal cost.

**Objective:**

The aim of this study was to evaluate the usability and acceptability of a theory-based Android mobile phone app for HIV prevention.

**Methods:**

The app included self-assessment tools; prevention recommendations; commodity (condoms, HIV self-tests) ordering; reminders to MSM for basic HIV prevention services, HIV testing, condom use, screening for preexposure prophylaxis (PrEP) and nonoccupational postexposure prophylaxis (nPEP); and prevention and treatment provider locators. The study recruited HIV-negative, Android-using MSM in Atlanta and Seattle who were asked to use the app for 4 months and complete a post-use survey. We measured the use of the app and its features, ordering of commodities, self-report of establishing an HIV testing plan, being HIV tested in the community, and starting PrEP or using nPEP. Usability was assessed using the system usability scale (SUS).

**Results:**

A total of 121 MSM were enrolled (59.5%, 72/121 from Atlanta; 40.5%, 49/121 from Seattle). Median age was 28. Nearly half (48.8%, 59/121) were nonwhite, and most (85.9%, 104/121) were gay-identified. Most had tested for HIV in the past (85.1%, 103/121), and 52 (43.0%, 52/121) had a plan to test for HIV regularly. Men used the app for an average of 17.7 minutes over the first 4 months. Over the 4-month period, over half ordered condoms (63.6%, 77/121) and HIV test kits (52.8%, 64/121) on the app. Eight of 86 (9%) PrEP-eligible MSM started PrEP during the 4-month period; of those, 6 of the 8 reported that the app influenced their decision to start PrEP. The mean SUS was 73 (above average).

**Conclusions:**

A theory-based mobile phone app was acceptable to MSM and was rated as having above-average usability. Most men used the commodity-ordering features of the app during the 4-month evaluation period, and nearly 1 in 10 PrEP-eligible men started PrEP, with most attributing their decision to start PrEP in part to the app. A broader, randomized controlled study of the impact of the app on uptake of prevention behaviors for MSM is warranted.

## Introduction

Human immunodeficiency virus (HIV) prevention has become an HIV sero-status-dependent practice, in which an HIV test is the first step toward either a prevention continuum for HIV-negative individuals, or a treatment and care continuum for those testing HIV-positive. In other words, HIV prevention must rest on a foundation of accurate knowledge of HIV sero-status among key populations, followed by sero-status-specific prevention approaches. For those who are HIV-negative, biomedical interventions such as preexposure prophylaxis (PrEP) hold promise to reduce susceptibility to HIV [1-3].

Men who have sex with men (MSM) are a key risk group in the United States and are disproportionately impacted in terms of HIV prevalence [4-6] and incidence [7-9]. MSM are the only US risk group for whom HIV incidence increased after 2000 [8]; increases are especially alarming among young (15-24 years old) MSM [7] and MSM of color [10]. This has resulted in profound health disparities for both MSM relative to other adult men and within the MSM community, with a burden of HIV infection that is a staggering 67 times greater than for other men in the US population [11]. Disparities are especially pronounced among MSM of color [4].

Multiple models of HIV incidence in MSM suggest that to decrease HIV incidence in MSM, we will need to achieve 30-50% coverage of multiple prevention services and interventions (eg, condom promotion, HIV testing, PrEP, treatment as prevention) in at-risk MSM [12-15]. However, the uptake of routine HIV testing and PrEP is low: less than half of MSM test for HIV yearly [16] and in 2013, <5% were utilizing PrEP [17]. A recent summary of electronic tools for HIV prevention in MSM noted that promotion of certain types of prevention services are most amenable to provision through new technologies. Services for which eligibility can be determined through an algorithm are good candidates to bring to scale with technologies [18]. For example, behavioral eligibility for PrEP has well-described criteria and eligibility algorithms [19,20]. Using technology to promote uptake of prevention services for MSM would also make services more accessible to rural MSM [18]. This is especially important because MSM in rural areas may have lower access to HIV prevention services delivered in community-based organizations [21,22].

Here, we present briefly the development of a comprehensive mobile HIV prevention app for MSM, and describe and report the initial evaluation of the app for usability and acceptability.

## Methods

### Previous Work and App Development

Needs assessment for an HIV prevention app for MSM was conducted prior to app development using a 3-phase, iterative process [23,24]. Phase 1 consisted of separate focus group discussions with MSM, HIV testing counselors, and key community informants to identify preferences and requirements to consider including in a mobile HIV prevention app. Preliminary data from phase 1 was used to build an alpha version of the app. The alpha version was then theater tested with additional focus group discussions. Data from all phases were then used to develop features, language, and security to build into the beta version of the app. All of the app development was completed by Keymind (McLean, VA), a technology firm that specializes in creating data systems and mobile apps, including apps for health care providers and systems. The authors (PS, RS, JS, and TG) were directly involved in creating the requirements for the app; Keymind staff produced the app.

### Theoretical Basis

The app content was developed based on the social cognitive theory of behavior [25]. Briefly, the app features enumerated in the following section were developed to fit into a framework of several health outcome behaviors (eg, making a plan to test regularly for HIV, using condoms, self-screening for PrEP, seeking HIV care for those living with HIV). For each health or prevention behavior, there were specific app features that were designed to promote goal setting, self-efficacy, outcome expectations, and self-regulation. For example, for the behaviors of HIV testing, the “Make a plan” app feature promoted goal setting, the presentation of several testing options and information promoted self-efficacy, information about the benefits of testing promoted positive outcome expectations, and a customizable reminder system for testing promoted self-regulation.

### App Features

A list of app features and descriptions are shown in Table 1; screenshots of the app are available in Multimedia Appendix 1. Highlighted features included monthly risk assessment quizzes that offered tailored HIV-prevention related recommendations, quizzes to self-assess PrEP and nonoccupational postexposure prophylaxis (nPEP) eligibility, resources to create and schedule a custom HIV testing plan with reminders, a global positioning system (GPS) enabled map of HIV testing locations with their operational details, and ordering of free condoms and at-home HIV test kits.

**Table 1:** Features of the HealthMindr app during the pilot study, United States, 2015.

Domain	Features
Initial and Monthly Risk Assessments	Provides tailored, HIV-related prevention suggestions for users to consider based on quiz responses. Monthly Assessments used responses from the previous month’s assessment to ask if there had been any changes to give up-to-date suggestions.
PrEP^a^ Screener	Assesses PrEP^a^ eligibility using seven questions developed by the CDC. The screener asks about time since last HIV^b^ test, number of partners in past 3 months, condom use frequency, partner’s HIV status, bacterial STIs^c^ in the past 12 months, and if engaged in exchange sex.
nPEP^d^ Screener	Assesses nPEP eligibility using a three question series about contact with bodily fluids, recency of exposure, and confidence in partners' HIV status [26].
Find My Frequency (HIV Testing)	Suggests HIV testing frequency of every 3 or 6 months based on five questions, including number of partners, partners' HIV status, bacterial STI infections in the last 12 months, and injection drug, meth, or poppers use [27,28].
Compare HIV Tests/ Help Me Choose	Allows users to prioritize the most important aspects of an HIV testing experience based on location type, sample collection method, cost, HIV counseling available, wait time for results, and window period of test. Users can filter tests based on their preferences or complete a quiz for recommendations based on their stated preferences.
My Test Plan	Users can plan an HIV test by date, time, and location. Automated reminders can be set based on a chosen testing frequency. After being tested, users can record their HIV/STI test results within the app to keep a record of testing history.
Reminders	Preferences can be set for how users receive testing and assessment reminders as pop-up notification, email, or neither. Users can choose the text of the reminder from a list of preset phrases or write their own message.
Ordering	Free at-home HIV test kits (OraQuick and Home Access), a variety condom styles, and silicone and water-based personal lubricants were offered.
Location Details & Map	Provides a map and details about testing locations, including address, phone number, type of organization, web address, days/hours of operation, service eligibility requirements (if any), fee information, languages available, and clinical services offered (HIV testing, HIV treatment, PrEP, nPEP, vaccinations, and so on). GPS^e^ was enabled to show user’s location relative to testing locations. Locations were able to be filtered by the above characteristics to display locations with select characteristics.
FAQs	Frequently asked questions related to HIV were included for users to reference. Users were also able to submit questions via the app to study staff.

^a^PrEP: preexposure prophylaxis.

^b^HIV: human immunodeficiency virus.

^c^STI: sexually transmitted infection.

^d^nPEP: nonoccupational postexposure prophylaxis.

^e^GPS: global positioning system.

### Pilot Study Overview

The purpose of this study was to assess the usability and acceptability of the HealthMindr app among MSM living in the metro areas of Atlanta, Georgia, and Seattle, Washington. The 2 cities were chosen because the availability of high-quality, gay-friendly prevention services differs in the 2 cities; we hypothesized that men who live in a city like Seattle where services are readily available and culturally competent might have less interest in accessing services through a mobile app. MSM were recruited on the Web and asked to install HealthMindr on their mobile phone, keep it on their phones for 4 months, and complete an evaluation survey at the end of the study period. Demographic and HIV prevention behaviors were collected during study enrollment. Brief periodic assessments were delivered monthly; the assessment of 10 risk questions allowed for prevention recommendations to be updated based on recent behaviors. App-based usage data was collected for all in-app actions participants made, including in-app button clicks, page views, and assessment or quiz responses. At the end of the participant’s study period, a Web-based evaluation survey was sent to participants to assess their HIV-related prevention behaviors during the pilot and app features that they did and did not find useful. Selected participants who were recommended to receive PrEP, including all who started PrEP, were invited to participate in individual in-depth interviews about their decision to start PrEP or not, and how the app influenced their decision-making process.

This study was approved by the institutional review boards of Emory University and the University of Washington. Participants were compensated US $25 each for completion of the baseline and 4-month follow-up surveys and US $5 each for the 3 periodic assessments administered through the app. Men who participated in individual in-depth interviewed were compensated US $40.

### Recruitment and Enrollment

Participants were recruited from May 2015 and August 2015 using advertisements on Facebook and a social or sexual networking mobile phone app for MSM. Advertisements targeted adult male Facebook users residing in Atlanta or Seattle who indicated being a man interested in men. Advertisements on the MSM networking app used geolocation to deliver advertisements to men who opened the app on an Android device while in the Atlanta or Seattle metro area.

Interested participants who clicked on an advertisement were taken to a Web-based screening and enrollment survey and presented with a brief description of the study. Men completed an electronic informed consent to be screened for study eligibility and then completed a brief screening survey; to be eligible for the study, participants must have been ≥ 18 years of age, English-speaking, living in the Atlanta or Seattle metropolitan areas, assigned male sex at birth, and identifying as male at the time of the screening; must have had sex with a man in the past year; must have never tested positive for HIV; and must have owned an Android mobile phone device with current service. Eligible men were asked to complete an electronic informed consent for study participation. Disqualified respondents were not given a reason for ineligibility and were provided the principal investigator’s contact information.

Consenting participants were next shown a 7-minute introductory video embedded within the enrollment survey (Multimedia Appendix 2). The video introduced study staff members, explained study procedures including compensation schedule, provided detailed instructions with app screenshots on how to download and register the app, and demonstrated different app features. Mobile phone ownership was verified by sending participants an SMS text message (short message service, SMS) with a confirmation number that the participant was required to enter into the enrollment survey before continuing. The last section of the enrollment survey asked for demographic information and HIV testing history during the previous 24 months, use of free condoms in the last year, and whether the participant had ever used PrEP, nPEP, or at-home HIV test kits.

Access to the app was limited to participants through the use of a unique registration code provided only to participants; password and personal identification number (PIN) protection were provided. After successfully registering the app, participants were asked to complete an initial in-app screening assessment, which completed their enrollment into the study. Enrollment completions that were suspected to be fraudulent based on duplicate or similar phone numbers, Internet protocol (IP) addresses, or email addresses were screened and verified by calling and speaking with the participant before accepting him into the study. In all cases, study staff called all study participants within a week of study enrollment to introduce themselves and answer any questions or concerns.

### Measures

#### Enrollment and Baseline Survey

Participants were asked demographic and baseline characteristics during study enrollment; including age in years; city of residence; race or ethnicity; sexual identity; recent HIV testing history; HIV status; plans for future HIV testing; and past use of PrEP, nPEP, condoms, and at-home HIV testing kits.

#### Evaluation Survey

After 4 months of use, participants were asked about motivation to use the app, HIV testing during the study period, PrEP and nPEP use during the study period, and at-home test kit and condom use for those who placed in-app orders. Participants were also asked to assess the app’s features, usability, design, content, and functionality using both Likert scales and optional open text fields. The usability of the app was further assessed using the system usability scale (SUS), a validated, industry standard scale used to evaluate a variety of products and services, including websites, mobile phones, computer software, and more [29]. The scale uses a series of questions to generate a usability score ranging from 0-100. An SUS score below 50 is not considered acceptable while above 70 is above average and >90 is superior [30].

### Analysis

The usage log was used to calculate the number of days participants used the app, pages of the app accessed, and the total time spent in the app. Time spent engaged within the app was quantified by calculating time passed between each action a user took and totaling the time for the visit. The longest 1% of time between actions (ie, longer than 2 minutes 38 seconds) was considered to not be representative of active app engagement. Time engaged within the app per person and per person-month was calculated. Descriptive statistics were used to examine app engagement and are reported as mean with range for time and action measurements. Participants’ ordering histories were kept for all at-home test kits, condoms, and personal lubricant orders placed. App pages accessed and features used by participants are reported as participant counts with percent. Evaluation responses are reported as percent of users who completed the evaluation survey. SUS results are reported as an aggregate score, using the method by which the scale was validated [30].

All analyses were performed using SAS 9.4 (SAS Institute Inc).

## Results

### Study Population

Of the 919 Web-based survey responses, 244 (26.5%, 244/919) left the survey after reading the study description, 108 (11.7%, 108/919) did not complete the screening survey, and 257 (28.0%, 257/919) did not meet eligibility requirements. Reasons for ineligibility included not owning an Android phone (42.8%, 110/257), being HIV positive (27.6%, 71/257), and living outside of the study area (18.3%, 47/257). Of the 309 eligible survey responses, 127 (41.1%, 127/309) did not complete the postscreening enrollment survey, 21 (6.7%, 21/309) completed the survey but did not download the app, and 40 (12.9%, 40/309) were determined to be fraudulent attempts to enroll multiple times and were disqualified. Final study enrollment was 121 MSM, including 72 in Atlanta and 49 in Seattle. App usage data were available for 90.0% (109/121) of participants. Ninety-eight (81.0%, 98/121) participants completed the 4-month evaluation survey. Participation in the evaluation survey did not differ by age (median test: *P*=.34); race (chi-square test: *P*=.90); or knowing of a local place to be tested for HIV (chi-square test: *P*=.99).

**Table 2:** Select baseline characteristics of men who have sex with men (MSM) participating in a 4-month pilot study of a human immunodeficiency virus (HIV) prevention app, United States, 2015.

Characteristic		Total (n=121)	Atlanta (n=72)	Seattle (n=49)
		n (%)	n (%)	n (%)
Male		121 (100)	72 (100)	49 (100)
Age in years, median IQR^a^		28 (24-34)	28 (24-35)	28 (23-33)
**Race or ethnicity**				
	White or Caucasian	62 (51.2)	34 (47.2)	28 (57.1)
	Black or African American	25 (20.7)	24 (33.3)	1 (2.0)
	Hispanic or Latino	10 (8.3)	3 (4.2)	7 (14.3)
	Asian or Pacific Islander	12 (9.9)	5 (6.9)	7 (14.3)
	Multiracial or other	12 (9.9)	6 (8.3)	6(12.2)
**Sexual orientation^b^**				
	Gay or homosexual	104 (86.0)	64 (88.9)	40 (81.6)
	Bisexual	14 (11.6)	8 (11.1)	6 (12.2)
**Times tested for HIV^c^** **in last 24 months**				
	0	12 (9.9)	8 (11.1)	4 (8.2)
	1-2	50 (41.3)	26 (36.1)	24 (49.0)
	3-4	32 (26.4)	23 (31.9)	9 (18.4)
	5+	27 (22.3)	15 (20.8)	12 (24.5)
**Most recent HIV test result**				
	Negative	103 (85.1)	58 (80.6)	45 (91.8)
	Never tested or unsure	18 (14.9)	14 (19.4)	4 (8.2)
**Knows local places to get an HIV test**				
	Yes	75 (62.0)	43 (59.7)	32 (65.3)
	No or don't know	24 (19.8)	14 (19.4)	10 (20.4)
	Did not answer	22 (18.2)	15 (20.8)	7 (14.3)
**PrEP^d^** **uptake**				
	Never used	106 (87.6)	65 (90.3)	41 (83.7)
	Previously used	4 (3.3)	3 (4.2)	1 (2.0)
	Currently use	11 (9.1)	4 (5.6)	7 (14.3)
**nPEP^e^** **usage**				
	Ever used	6 (5.0)	5 (6.9)	1 (2.0)
**Free condoms in last 12 months**				
	Received and used	55 (45.5)	33 (45.8)	22 (44.9)
	Received and did not use	21 (17.4)	12 (16.7)	9 (18.4)
**At-home HIV test**				
	Ever used	33 (27.3)	14 (19.4)	19 (38.8)

^a^IQR: interquartile range.

^b^1 missing, 1 pansexual, 1 queer for Seattle.

^c^HIV: human immunodeficiency virus.

^d^PrEP: preexposure prophylaxis.

^e^nPEP: nonoccupational postexposure prophylaxis.

Select baseline characteristics of participants are shown in Table 2. The median age of participants was 28 years and did not differ between Atlanta and Seattle. Nearly half of study participants were nonwhite; Seattle had a higher proportion of white participants than Atlanta. Nearly two-thirds knew of a local location to get an HIV test. During the previous 24 months, almost half of participants had tested for HIV 3 or more times; 15.7% (19/121) had never been tested or were unsure of their HIV status. Over 1 in 10 participants had used PrEP before; reported PrEP usage (ever) was higher in Seattle (16%, 8/49) than in Atlanta (10%, 7/72%).

### App Engagement

Participants’ app engagement is presented in Table 3. Over 4 months, participants used the app on average for 17 minutes 40 seconds and made 133 clicks. There were no differences by median test between participants from Atlanta and Seattle in terms of total time spent on the app (*P*=.91) or total clicks (*P*=.62). There were also no differences in total time spent on the app by age (*P*=.21), race or ethnicity (*P*=.65), or whether participants knew of a local place to be tested for HIV (*P*=.99).

Total engaged time ranged from 25 seconds up to 77 minutes. Typically, the first visit was the longest (average first visit time: 7 minutes). Although the number of participants using the app each month declined, returning participants continued to engage with the app consistently with engagement during months 2-4 averaging 6.5 minutes and 49 clicks per month among active users. Most participants returned to the app multiple times over the 4-month period: 35% used the app on between 2 and 4 days, and 42% used the app on 5 or more days. Participants averaged using the app on a mean of 4.9 days.

**Table 3:** Time engaged and user clicks made in a human immunodeficiency virus (HIV) prevention app by men who have sex with men (MSM) participants during 4-month pilot study, United States, 2015.

Criterion			Time engaged		Clicks^a^	
**Usage**		n	Average (minutes) per user	Range	Average per user	Range
	Total pilot usage	109	17.7	0.4-76.8	133	7-572
	First visit usage	109	7.0	0.4-22.2	52	7-131
**Participant no.**						
	1	109	11.3	0.4-61.2	85	7-454
	2	47	6.2	1.3-19.6	46	15-184
	3	35	5.7	0.2-20.3	46	2-118
	4	25	8.2	0.8-35.2	61	11-191

^a^Clicks capture all single actions made by a user, including logins, button clicks, and app navigation.

**Figure 1:**
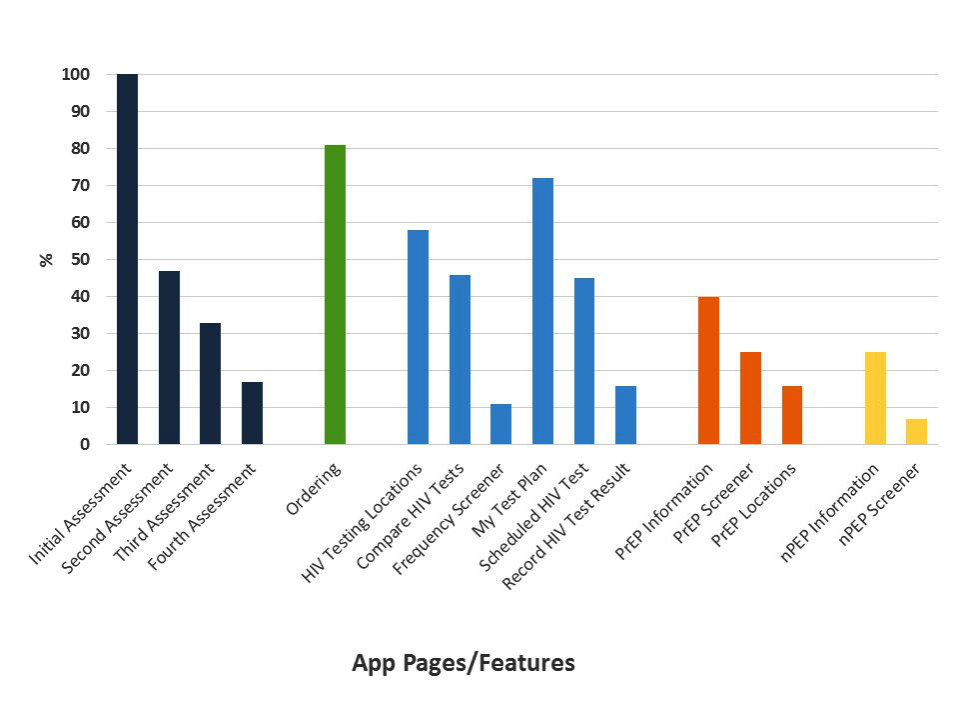
Percent of MSM participants that used features or viewed pages in the app during a 4-month pilot study, United States, 2015 (n=109). MSM: men who have sex with men.

The percent of participants that used app features are reported in Figure 1. All participants completed the initial assessment, a requirement for enrollment into the study. Fewer participants completed the monthly assessments. HIV testing features were frequently accessed; ordering test kits and condoms was the most frequently accessed feature. Forty percent viewed PrEP information, a quarter used the PrEP screener, and nearly 1 in 5 opened the provider map feature from their PrEP screener result to view locations that offered PrEP. nPEP information was accessed by 25% of participants and 7% screened themselves for nPEP eligibility at least once.

### Ordering

Orders placed for free condoms and at-home HIV test kits are summarized in Table 4. Nearly two-thirds of men ordered condoms and over half ordered an at-home HIV test kit at least once. Of 154 kits ordered, most (84%, 129/154) were OraQuick kits. Most who placed an order did so on their first visit. Many participants placed multiple orders for condoms (38% of all who ordered) and for at-home HIV test kits (41%). Most at-home HIV test kits received were used by the participants to test themselves; 10% gave their test kit to someone else. Test kit use varied between the cities; 12% of Atlanta participants did not use the test kit compared with 41% of Seattle participants.

**Table 4:** Condoms and at-home human immunodeficiency virus (HIV) test kit ordered from an HIV prevention app by men who have sex with men (MSM) participants during a 4-month pilot study, United States, 2015.

Characteristic		Total (n=121) n (%)	Atlanta (n=72) n (%)	Seattle (n=49) n (%)
**Condom orders^a^**				
	On 1st visit	64 (52.9)	38 (52.8)	26 (53.1)
	At least once during pilot	77 (63.6)	47 (65.3)	30 (61.2)
	Place repeat order	29 (24.0)	16 (22.2)	13 (26.5)
**At-home HIV test orders^a^**				
	On 1st visit	37 (30.6)	28 (38.9)	9 (18.4)
	At least once during pilot	64 (52.9)	39 (54.2)	27 (55.1)
	Placed a repeat order	26 (21.5)	18 (25.0)	8 (16.0)
		n=70	n=45	n=25
**Used the ordered condoms^b^**				
	Yes	61 (87.1)	40 (88.9)	21 (84.0)
	No	9 (12.9)	5 (11.1)	4 (16.0)
**Condoms replaced condoms would have bought or received elsewhere^b^**				
	Yes	51 (72.9)	31 (68.9)	20 (80.0)
	No	18 (25.7)	14 (31.1)	4 (16.0)
**Had condoms at time of order^b^**				
	Yes	40 (57.1)	20 (44.4)	20 (80.0)
	No	30 (42.9)	25 (55.6)	5 (20.0)
		n=50	n=33	n=17
**Use of human immunodeficiency virus (HIV) home test^b^**				
	Self	34 (68.0)	25 (75.8)	9 (52.9)
	Significant other	3 (6.0)	3 (9.1)	0 (0.0)
	Friend	1 (2.0)	1 (3.0)	0 (0.0)
	Acquaintance	1 (2.0)	0 (0.0)	1 (5.9)
	Not yet used	11 (22.0)	4 (12.1)	7 (41.2)
**Prior intentions of at-home HIV test users^b^**				
	Not planning to be tested	34 (68.0)	21 (63.6)	13 (76.5)
	Replaced a planned test	16 (32.0)	12 (36.4)	4 (23.5)

^a^Order history analyses include all pilot participants (n=121).

^b^Reported in final evaluation survey (n=98).

Of those who ordered condoms, 87.1% reported using them. When asked about their motivations to place a condom order, participants said it was because the condoms were free (76%), it was convenient to do so in the app (67%), and they wanted to try different condom types (66%). Over two-thirds of participants who ordered test kits said they did not plan on being tested for HIV but ordered an at-home HIV test kit because it was offered in the app.

### Outcomes

HIV and sexually transmitted infection (STI) testing behaviors during the pilot study are also shown in Table 5. Almost 80% of participants tested for HIV at least once during the pilot, and 56% tested multiple times. Additionally, almost half were screened for STIs. Three Atlanta participants tested newly positive for HIV. Their ages ranged from 18-29 years; 1 had never been tested for HIV before, and the other 2 had been tested for HIV 3 and 10 months before their enrollment. One participant had ordered and received an HIV test kit from the study. Two of the 3 who tested positive said their main motivation for being tested for HIV was the recommendation of the app to be tested; one said he tested routinely and had planned the test regardless of the app recommendation. Among participants who did not have a set HIV testing schedule at the start of the pilot, 63% had a schedule at the end of the pilot.

**Table 5:** Human immunodeficiency virus (HIV) and sexually transmitted infection (STI) testing history of men who have sex with men (MSM) participants during a 4-month pilot study of an HIV prevention app, United States, 2015.

Health Behavior at Post-Use Survey		Total (n=98)	Atlanta (n=61)	Seattle (n=37)
		n (%)	n (%)	n (%)
**HIV/STI Testing**				
	Tested for HIV^a^	75 (77)	48 (79)	27 (73)
	Tested HIV positive	3 (4)	3 (6)	0 (0)
	Tested for STIs^b^	46 (47)	29 (48)	17 (47)
**HIV Test Plan**				
	Had a previous HIV testing plan	52 (53)	34 (56)	18 (49)
	Did not have a previous plan, but now does	29 (30)	18 (30)	11 (30)
	Does not have an HIV testing plan	17 (17)	9 (15)	8 (19)
				
**Among those never tested or tested > 1 year ago at baseline**		n=19	n=12	n=7
	Tested during pilot	13 (68)	8 (67)	5 (71)
	Tested HIV positive	1 (5)	1 (8)	0 (0)
**HIV Test Plan**				
	Had a previous HIV testing plan	6 (32)	4 (33)	2 (29)
	Did not have a previous plan, but now does	9 (47)	7 (58)	2 (29)
	Does not have an HIV testing plan	4 (21)	1 (8)	3 (43)

^a^HIV: human immunodeficiency virus.

^b^STIs: sexually transmitted infections.

At the beginning of the study, 24% of participants reported not having heard of PrEP and 53% reported not knowing about nPEP. During the app pilot, 9% (8/86) of PrEP-eligible participants not already taking PrEP began taking PrEP, and 1 participant used nPEP. Among the 8 men who started PrEP, 6 reported that the app influenced their decision to start PrEP for one or more reasons (because the participant did not know what PrEP was before using the app [1/8]; because the app recommended PrEP based on behavioral assessments [1/8]; because the app provided information about PrEP [3/8]; because the app allowed them to find a PrEP provider [3/8]).

### App Evaluation

The content of the app was thought of positively overall, with 88% finding the level of detail and 81% finding the assessment recommendations to be useful or very useful. Additionally, 66% felt the app content helped them to stick to an HIV prevention plan. Most participants felt the app was a good balance of personal and professional language (71%) and the information was easy to understand (90%). Most participants felt confident in app security (86%), including a password or PIN offering sufficient protection (85%) and the app name and icon not readily associated as an HIV prevention app (84%).

The usability of the app was well received by participants with findings shown in Table 6. Overall, participants found the app to be easy to use and felt confident that they would be able to learn how to use it quickly and without technical assistance. The average composite score for the app was 73.4 (above average) [30].

**Table 6:** System usability scale (SUS) scores of an human immunodeficiency virus (HIV) prevention app by men who have sex with men (MSM) participants during 4-month pilot study, United States, 2015 (n=98).

Statement	Mean^a^ (SD)	Absolute^b^
I would like to use this app frequently.	3.7 (SD 1.1)	3.7
The app was unnecessarily complex.	2.2 (SD 1.0)	3.8
The app was easy to use.	4.1 (SD 0.8)	4.1
I would need support from a technical person to be able to use this app.	1.8 (SD 1.0)	4.3
Various functions in the app were well integrated.	3.9 (SD 1.0)	3.9
There was too much inconsistency in this app.	2.1 (SD 0.9)	3.9
Most people would learn to use this app very quickly.	4.1 (SD 1.0)	4.1
The app was very cumbersome to use.	2.4 (SD 1.0)	3.6
I felt very confident using the app.	4.0 (SD 0.8)	4.0
I had to learn many things before I could get going with this app.	2.0 (SD 1.0)	4.0
Calculated score	73.4 (SD 16.7)	

^a^Scoring based on a scale from 1=totally disagree to 5=totally agree.

^b^Adjusts scores of negative statements so larger numbers are associated with positive statements.

When participants were asked about future app use, most said they would probably or definitely download the app again (69%), recommend it to a friend (71%), and continue to use it as part of their HIV prevention plan (66%). Very few participants reported they would probably or definitely not download it again (5%), not recommend the app to a friend (3%), or not continue to use the app themselves (13%).

## Discussion

### Principal Findings

There are many reasons to be excited about the use of mobile apps to increase the uptake of basic HIV prevention services among MSM. Achieving our national strategy goal of reducing new HIV infections by 25% by 2020 will require making substantial improvements in HIV prevention for MSM. Based on current use of HIV prevention services [31], achieving the 30%-50% coverage of basic prevention services that will likely be needed to achieve such reductions would require either broad expansions of funding for existing service provision mechanisms or development of new modes of delivery that can scale with low incremental costs [18]. And the very communities of MSM most impacted by ongoing transmissions—young MSM and MSM of color—are the same men most likely to have mobile phones [32]. In short, there is an intersection of public health need, good fit of technology to programmatic needs, and high coverage of required device ownership among those at greatest need of services. Men in rural areas of the United States are in equal need of HIV prevention services, but are less likely to report receiving HIV testing and condom distribution, and are less likely to be aware of PrEP or have accessed PrEP [17]. Our data indicate that men will use a mobile phone app for comprehensive HIV prevention services, including engagement in HIV testing, condom use, and PrEP assessment, and that such an app is acceptable to MSM at risk for HIV acquisition.

Various metrics have been proposed to characterize engagement with mobile phone apps [ [33], although there are very limited data specific to health-related apps. With respect to engagement in the app, our data indicate that there was substantial variation in the extent of engagement with the app. Overall usage time was about 1.5 minutes per week over the period, which suggests that usage of our app was above average (above the median level of engagement observed in a recent analysis of usage of 22,000 mobile phone apps by over 600,000 mobile phone users) [33]. Our user database from the trial participants, 121 users, places our app above the 80^th^ percentile in terms of numbers of users of available apps [33]. Thus, even in this limited implementation, our app shows engagement characteristics that are more favorable than most available apps.

Another way to measure engagement is the ordering and use of commodities by app users. Most users ordered condoms, HIV test kits, or both. It is also significant that most users who ordered condoms or HIV test kits reported using them. In a survey of MSM who received free condoms in bar or club settings, less than three quarters reported using the condoms they received [34]; in our study, nearly 90% of those who ordered condoms used them. It is important to recognize that many health jurisdictions already provide distribution of free condoms, sexual lubricants, and HIV test kits. In most places, this is accomplished through paying staff to distribute commodities in high risk venues. Distributing these prevention commodities by mail to those who want them and order them might offer a more targeted and cost-efficient way to fulfill public health functions of condom distribution and HIV testing promotion.

PrEP is an emerging biomedical approach to reducing HIV acquisition risk in high-risk MSM, but uptake of PrEP among MSM has been slow. Levels of awareness of PrEP are also low, especially among younger MSM and MSM in rural areas [17]. When less than 5% of US MSM have ever used PrEP, it is striking that nearly 10% of PrEP-eligible men initiated PrEP within a 4-month period of app use. According to our theoretical model of the PrEP continuum [35] and our qualitative data, the app likely promoted PrEP uptake by increasing awareness of PrEP (PrEP information features), facilitating access to PrEP providers, and helping men identify their personal risk of HIV infection and indications for PrEP. We recognize that some additional information such as information on costs and clinical procedures are currently lacking from the app, and based on the feedback we received from the participants, we will develop and add new materials to the PrEP component of the app.

According to a broadly accepted standard assessment of usability, our app was assessed as being above average using traditional criteria [30]; using a validated translation to qualitative terms, our app would be assessed as being between good and excellent [36]. Our data also suggest the possibility of social diffusion of the app, when it is made more broadly available: over 7 in 10 participants reported being willing to recommend the app to a friend. MSM have reported that recommendation from a trusted source was a major factor influencing their willingness to use an app [37]. By these standards, we conclude that our app is acceptable to the MSM whom we would have use the app.

### Limitations

Our study had several limitations. First, our participants were subject to selection bias across several dimensions. We recruited men who were using Facebook or a Web-based dating app, and who may have been more comfortable using mobile apps than other men. We restricted our study to MSM in Seattle and Atlanta because of the need to provide enhanced resource directories; men in other cities might view and use the app differently. We limited this evaluation to men whose phones used the Android operating system; users of Apple or Windows operating system (OS) phones might have different use experiences or opinions of the app. However, we note that Android phone ownership is higher among Americans of color and among younger Americans, who are the groups with the highest rates of new HIV infections [32]. Our usage might have been overestimated because participants were enrolled in a research study with compensation for study assessments. Our pilot occurred only among MSM in urban areas, so generalizability to other MSM (including those in rural areas) was limited. Although nearly half of our sample was nonwhite, we had limited enrollment of Hispanic MSM. Enrolling racial or ethnic minorities in Web-based prevention research has been historically challenging, and future studies should over-recruit MSM of color, including Hispanic MSM [38].

There is a broad interest in the use of mobile apps for HIV prevention and a scientific evidence base to support the idea that mobile apps can influence health behaviors. We have developed a theory-based mobile phone app to provide a basic package of HIV prevention services to MSM, and found it to be acceptable to users in Seattle and Atlanta. Furthermore, our data on usage of specific components and order of commodities provide examples of how engaging with the app could improve health outcomes and provide baseline estimates of uptake, which can be used to power future randomized studies of the app. We recommend that, because of the high costs of app development, prevention scientists use a staged approach of qualitative formative work, theater testing, and usability or acceptability testing to ensure that mobile apps that are moved into larger, more expensive efficacy trials meet basic standards of acceptability and usability. The HealthMindr app has been demonstrated as being acceptable to MSM, as being usable, and as being associated with use of prevention services. HealthMindr should be considered for further evaluation in a randomized controlled trial with outcomes of the uptake of prevention behaviors.

## Appendix

Multimedia Appendix 1 Screenshots of HealthMindr app.

Multimedia Appendix 2 Welcome video provided to participants to introduce the study and procedures.

## Abbreviations

AIDS: acquired immunodeficiency syndrome
GPS: global positioning system
HIV: human immunodeficiency virus
MSM: men who have sex with men
nPEP: nonoccupational postexposure prophylaxis
PrEP: preexposure prophylaxis
PIN: personal identification number
STI: sexually transmitted infection
SUS: system usability scale
