# Supplementary material for: Usability and Acceptability of a Mobile Comprehensive HIV Prevention App for Men Who Have Sex With Men: A Pilot Study
Source: JMIR Mhealth Uhealth. 2017 Mar 9;5(3):e26. doi: 10.2196/mhealth.7199 (PMC5364322; doi:10.2196/mhealth.7199)
Supplement: Multimedia Appendix 1 [file mhealth_v5i3e26_app1.pdf]

Appendix. Screenshots of HealthMindr app for Android OS.

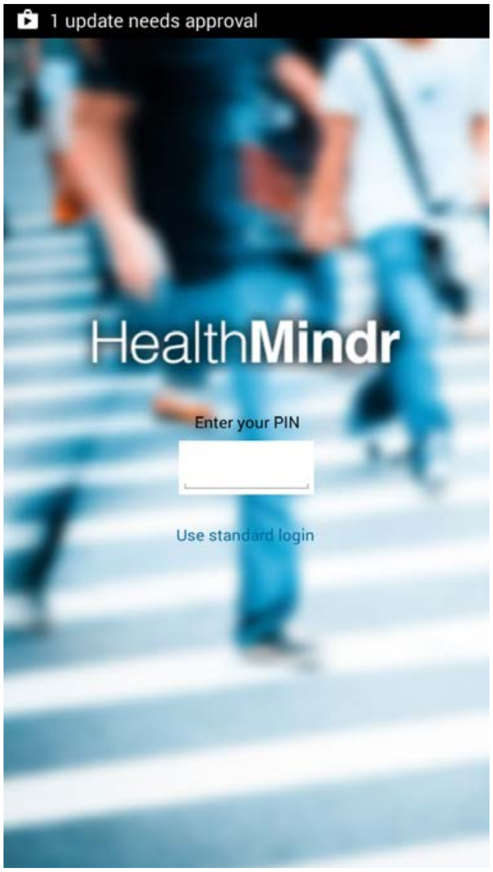

Figure A1. HealthMindr login screen.

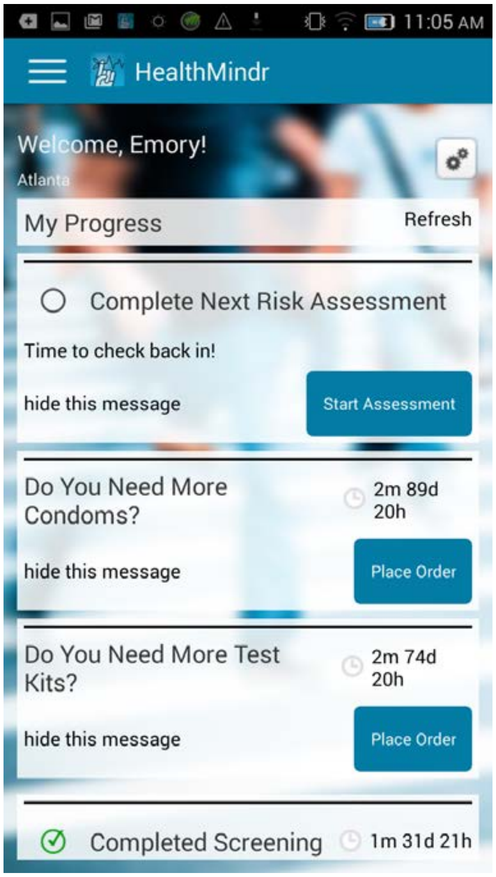

Figure A2. HealthMindr welcome screen.

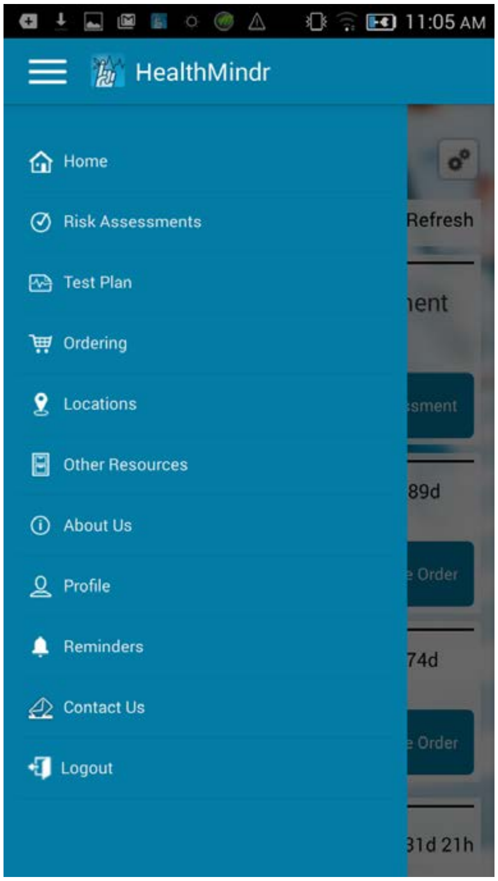

Figure A3. HealthMindr app menu.

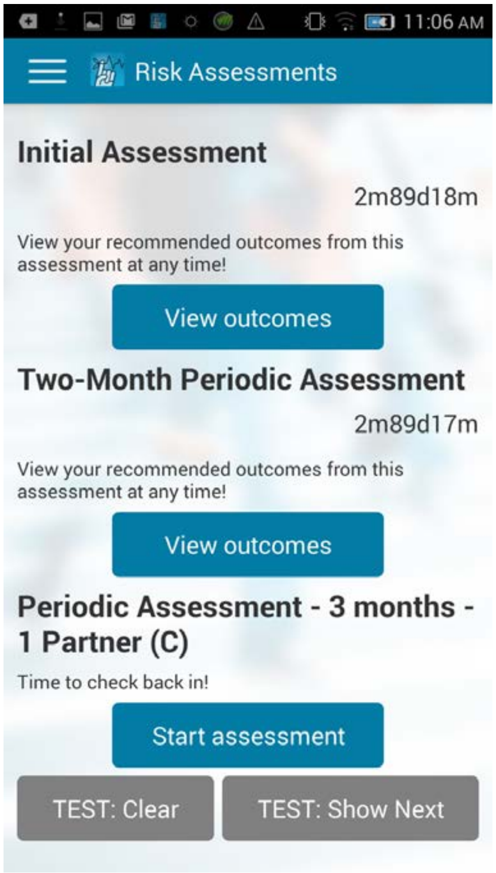

Figure A4. Risk assessments menu.

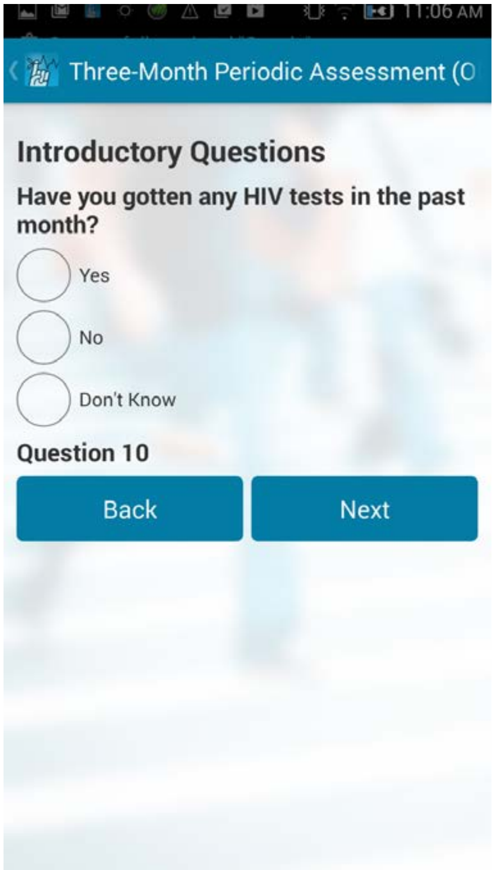

Figure A5. Periodic self-assessment.

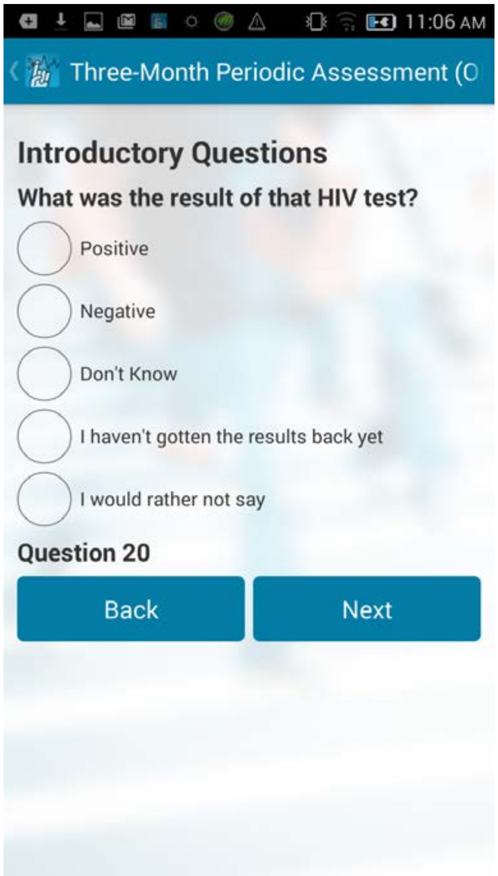

Figure A6. Periodic self-assessment.

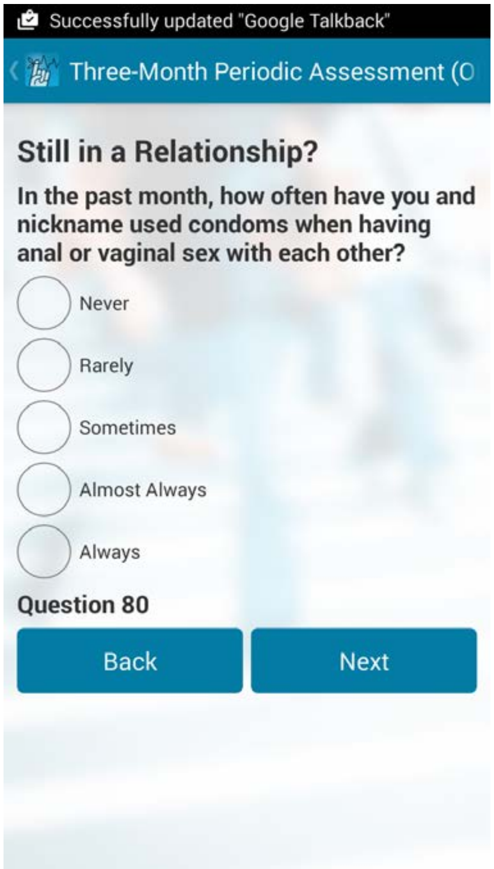

Figure A7. Periodic self-assessment.

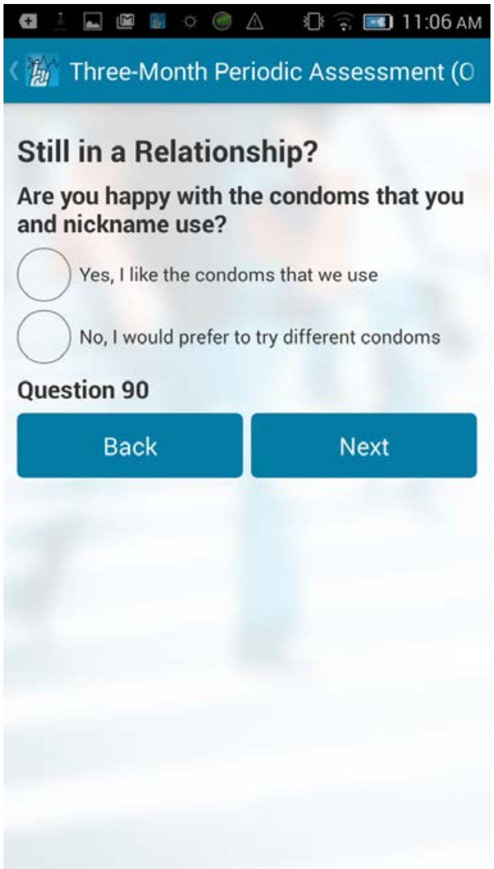

Figure A8. Periodic self-assessment.

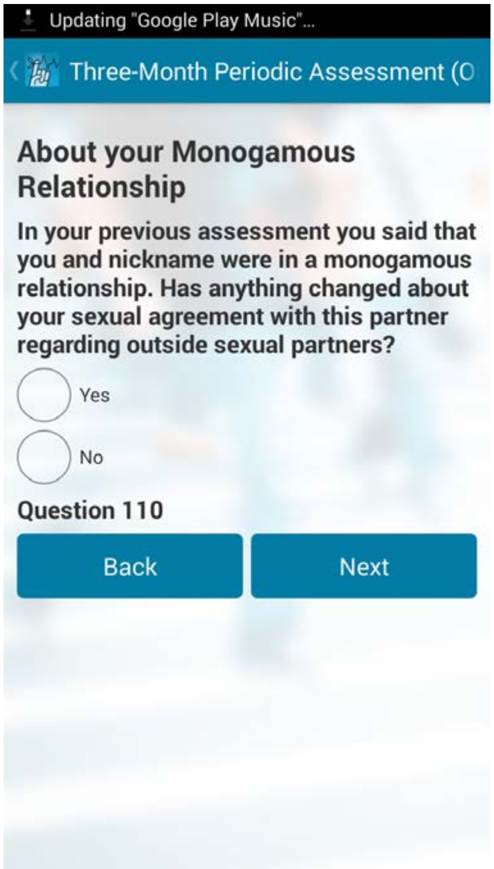

Figure A9. Periodic self-assessment.

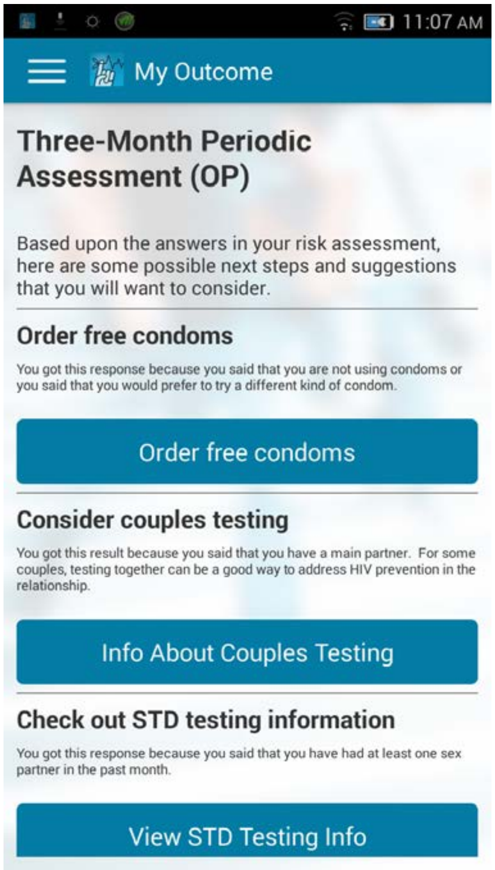

Figure A10. Periodic self-assessment outcomes.

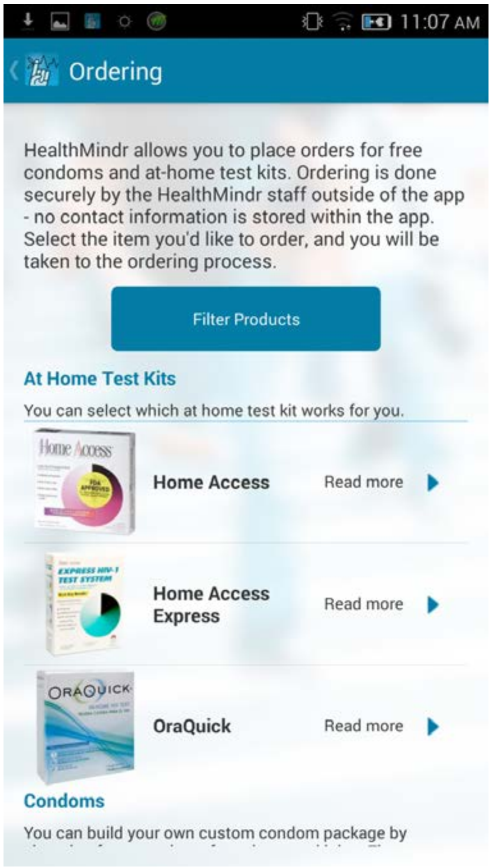

Figure A11. At home test kit ordering.

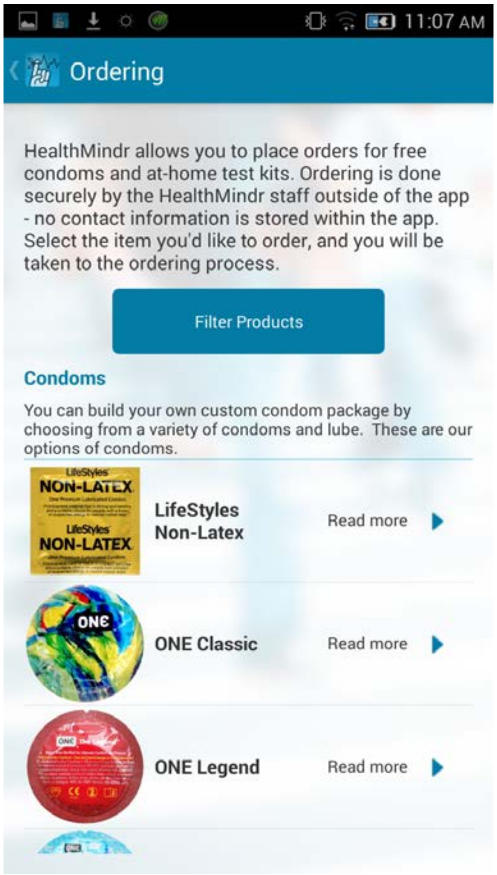

Figure A12. Condom ordering.

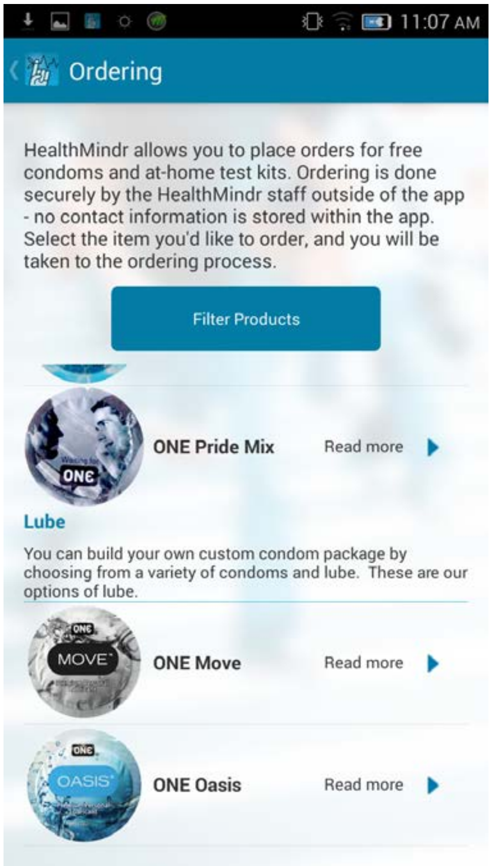

Figure A13. Condom-compatible lubricant ordering.

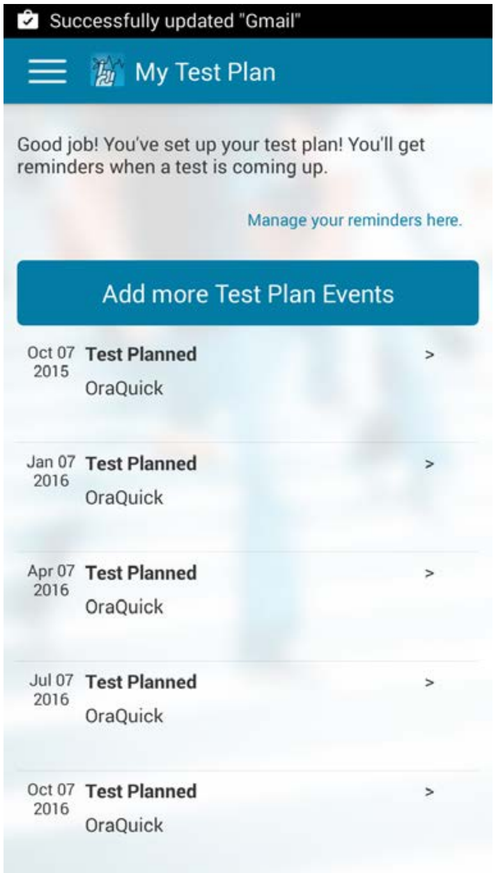

Figure A14. Test plan page.

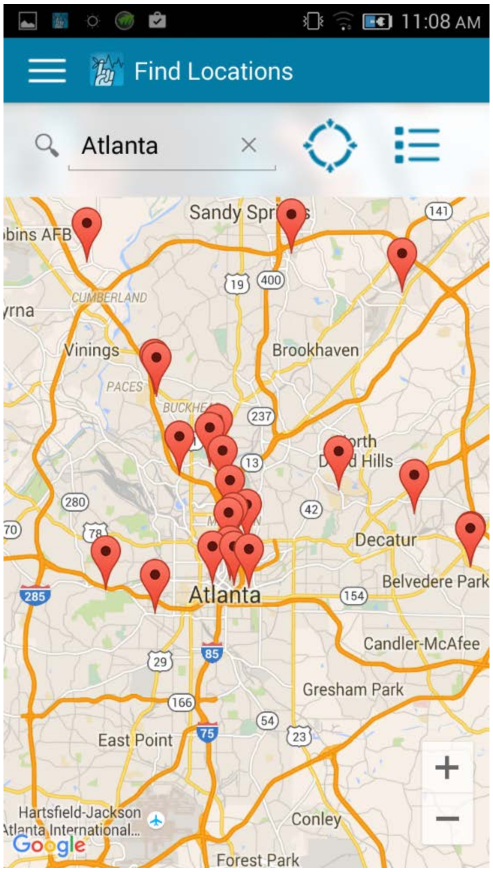

Figure A15. HIV testing locations map.

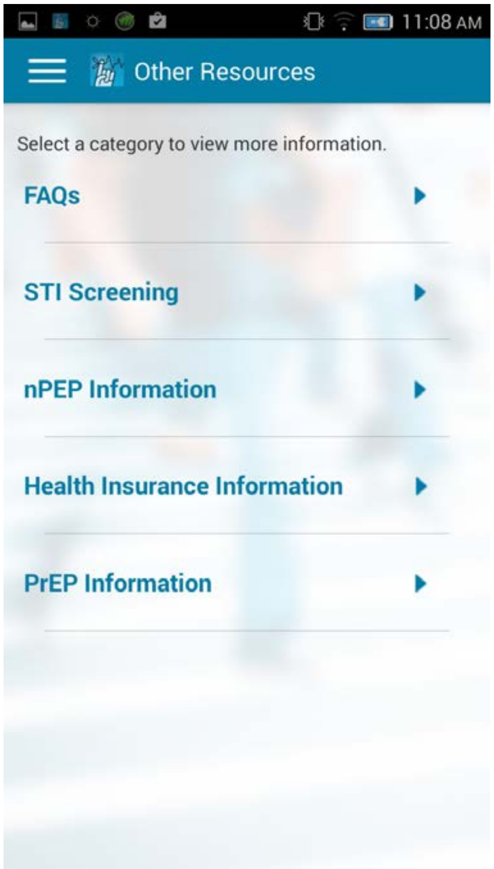

Figure A16. Other resources.

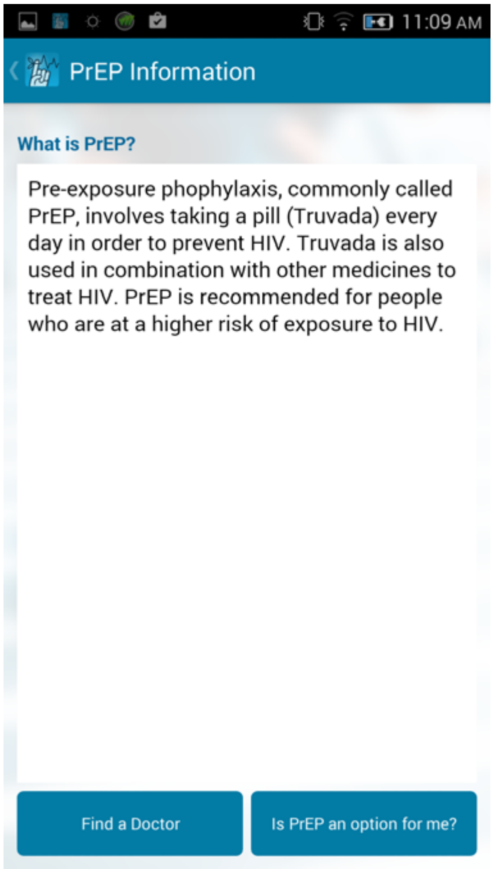

Figure A17. PrEP information.

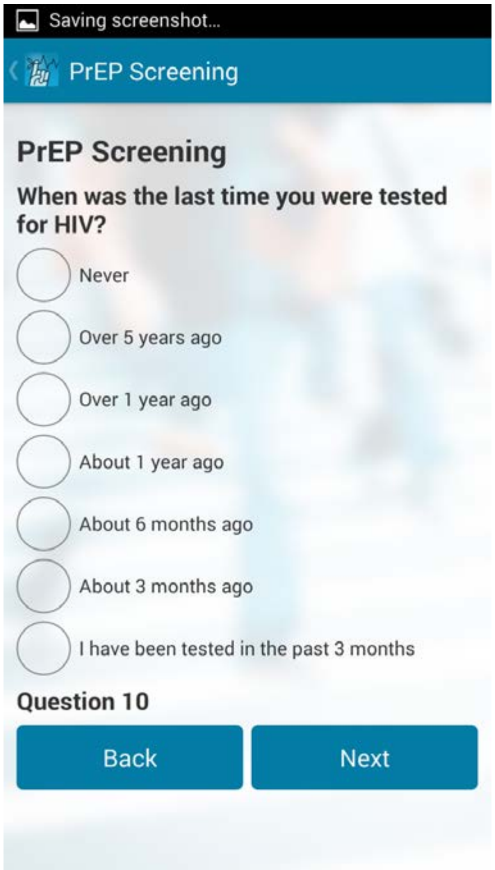

Figure A18. PrEP self-assessment.

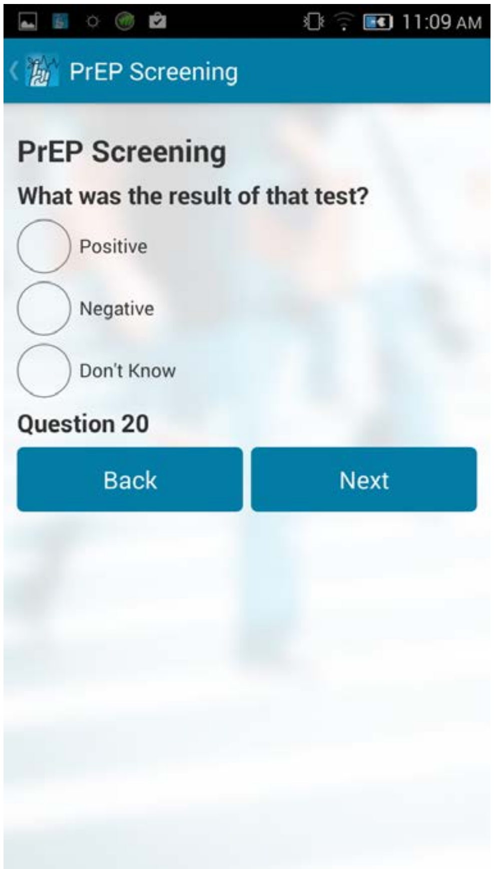

Figure A19. PrEP self-assessment.

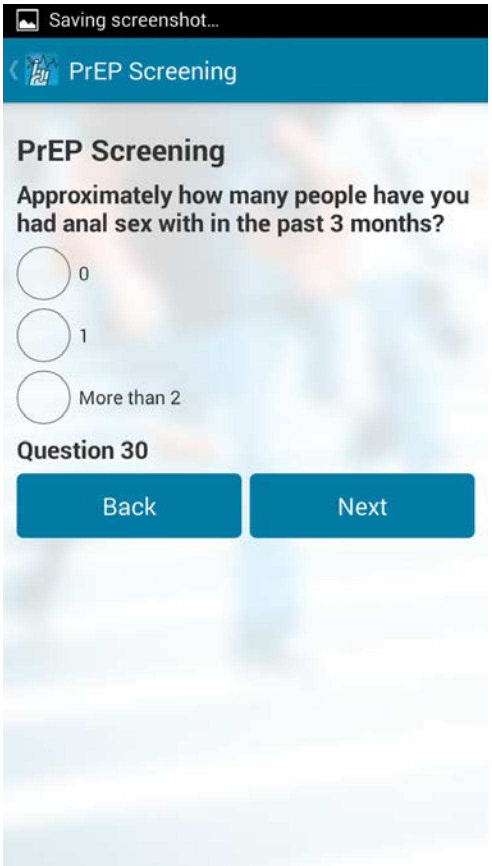

Figure A20. PrEP self-assessment.

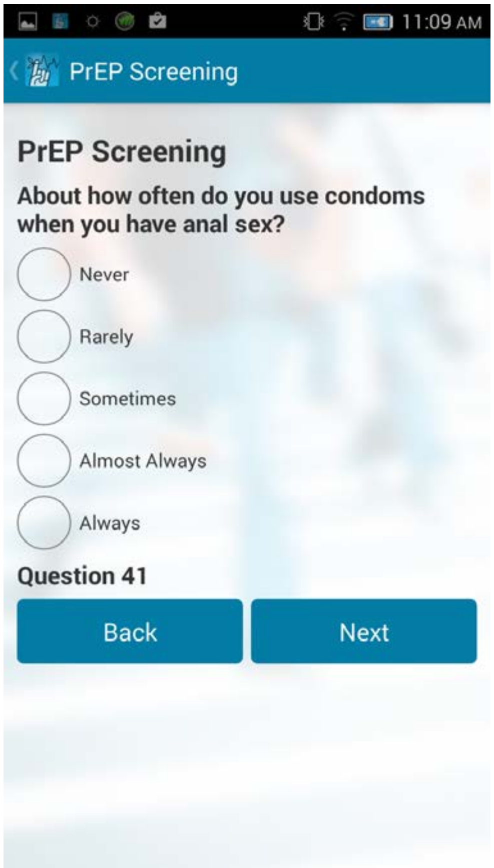

Figure A21. PrEP self-assessment.

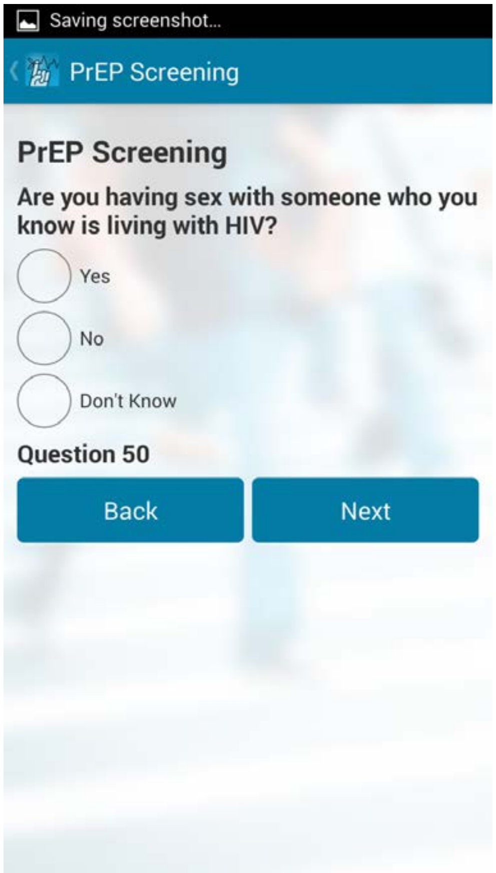

Figure A22. PrEP self-assessment.

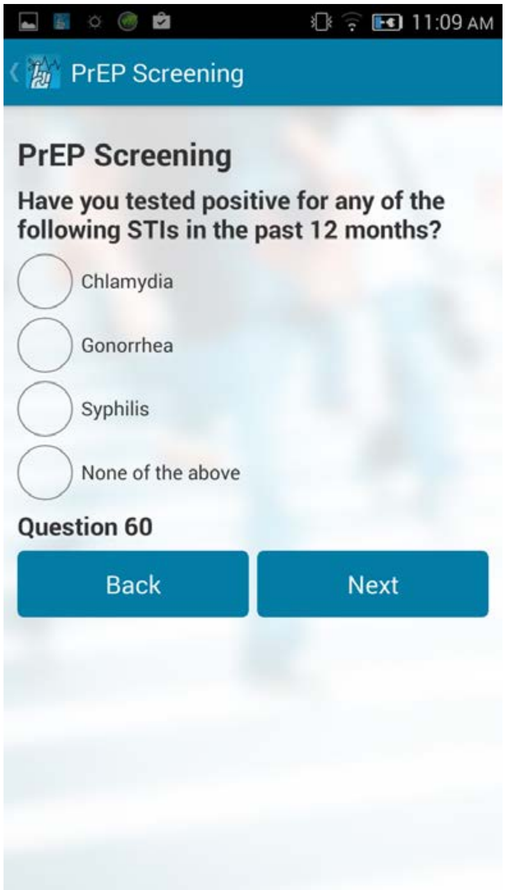

Figure A23. PrEP self-assessment.

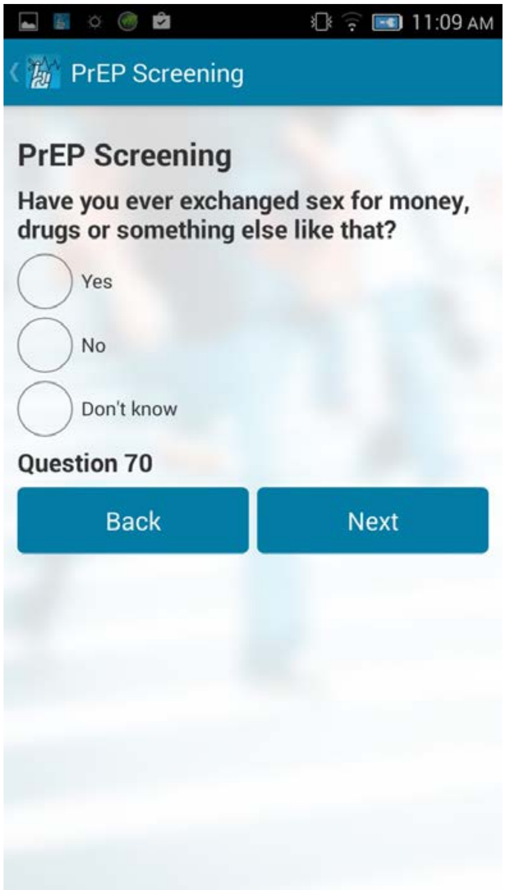

Figure A24. PrEP self-assessment.

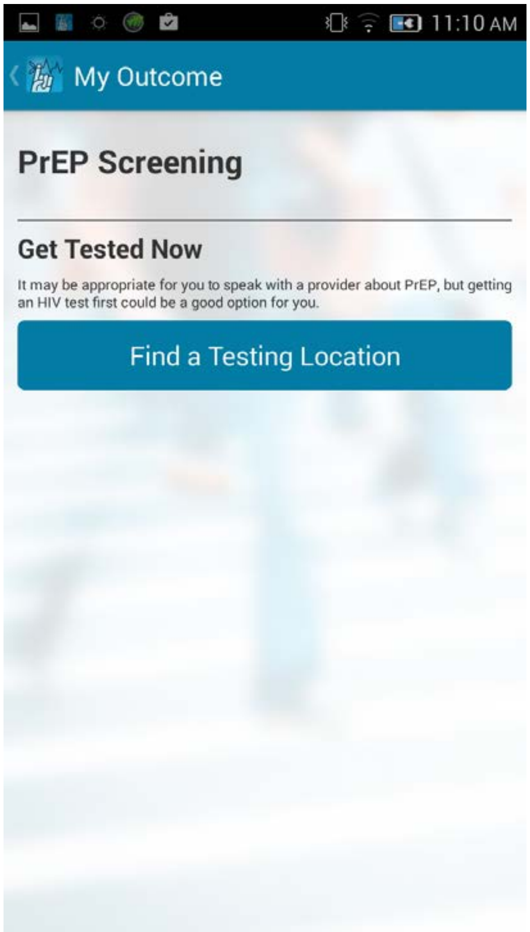

Figure A25. PrEP self-assessment results - recommend testing first.

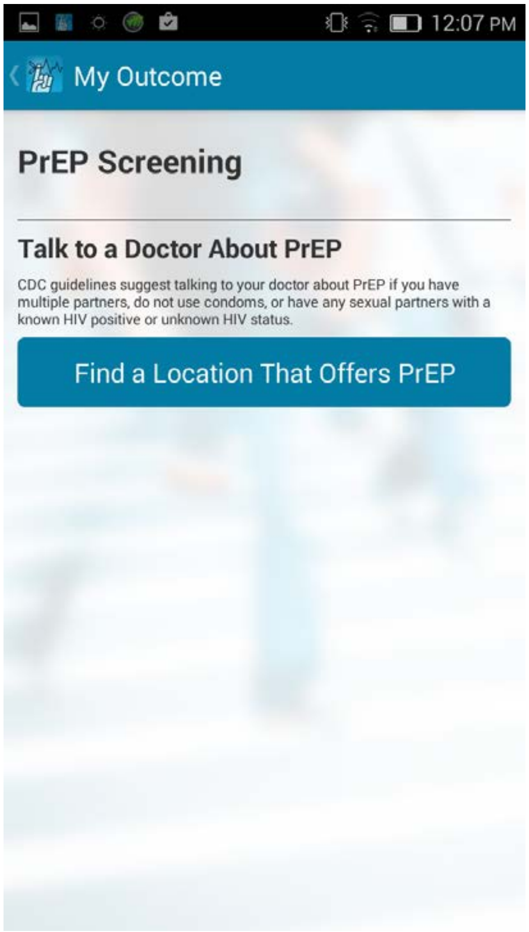

Figure A26. PrEP self-assessment results -. recommend talking to a doctor about PrEP.

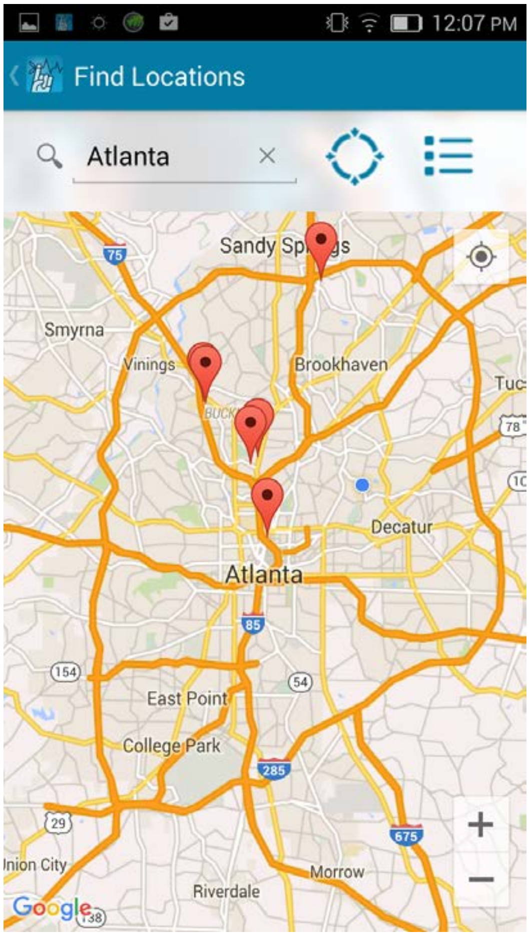

Figure A27. PrEP provider locations map.

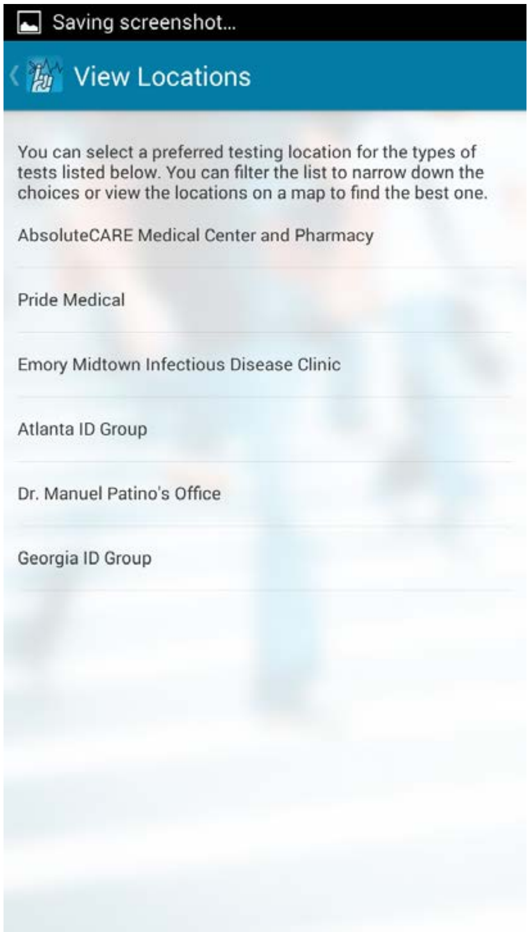

Figure A28. PrEP provider locations list.

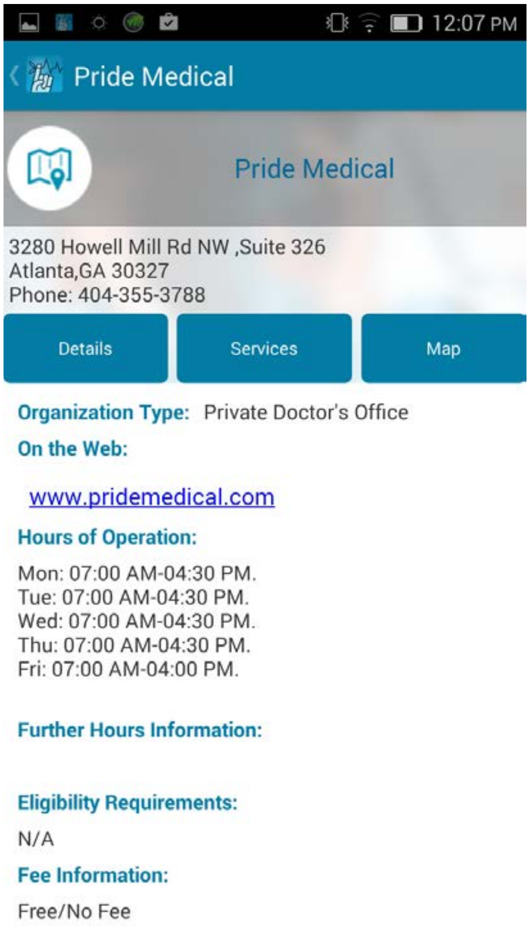

Figure A29. Detailed provider information.

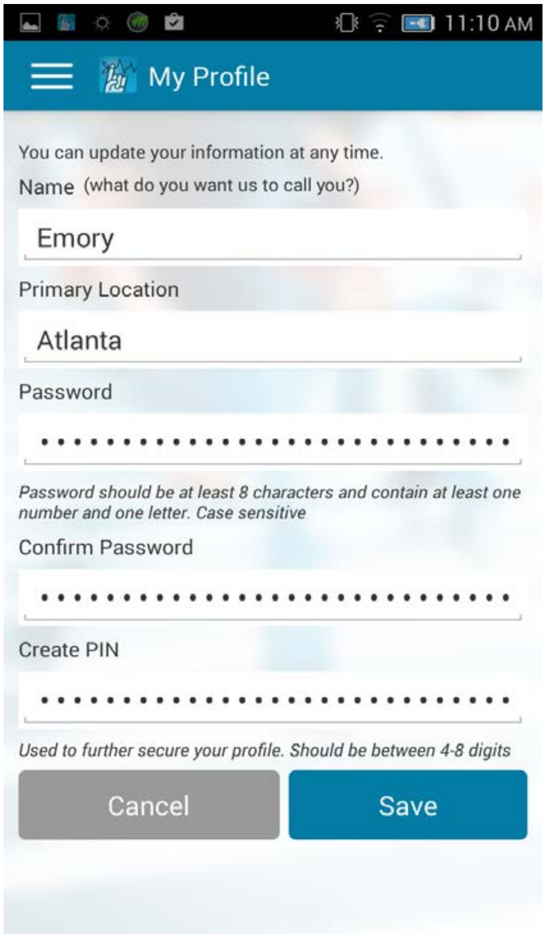

Figure A30. HealthMindr profile page.

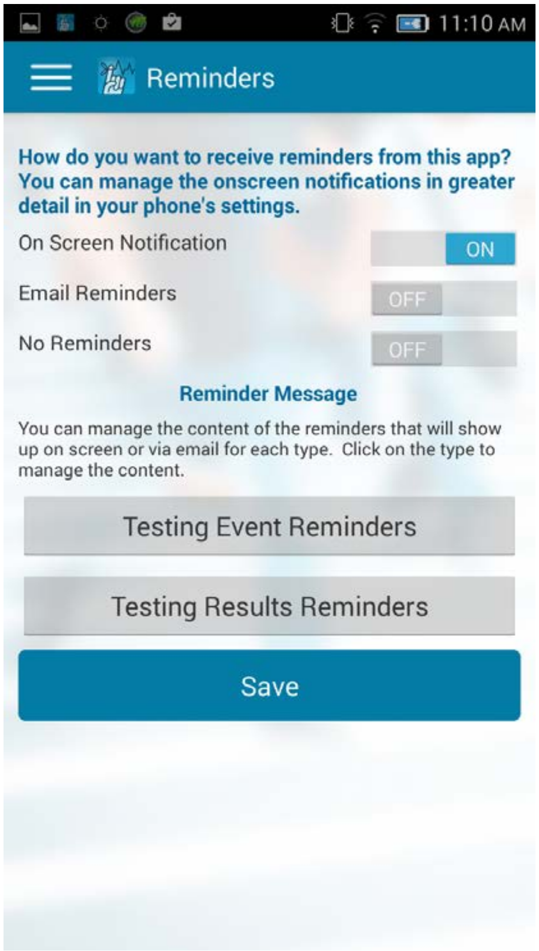

Figure A31. Reminders page.
